# Emerging fungal infections among children: A review on its clinical manifestations, diagnosis, and prevention

**DOI:** 10.4103/0975-7406.72131

**Published:** 2010

**Authors:** Akansha Jain, Shubham Jain, Swati Rawat

**Affiliations:** SAFE Institute of Pharmacy, Gram Kanadiya, Indore, India; 1 Shri Bhagwan College of Pharmacy, Aurangabad, India

**Keywords:** Candida diaper rash, histoplasmosis, opportunistic, sporotrichosis

## Abstract

The incidence of fungal infections is increasing at an alarming rate, presenting an enormous challenge to healthcare professionals. This increase is directly related to the growing population of immunocompromised individuals especially children resulting from changes in medical practice such as the use of intensive chemotherapy and immunosuppressive drugs. Although healthy children have strong natural immunity against fungal infections, then also fungal infection among children are increasing very fast. Virtually not all fungi are pathogenic and their infection is opportunistic. Fungi can occur in the form of yeast, mould, and dimorph. In children fungi can cause superficial infection, i.e., on skin, nails, and hair like oral thrush, candida diaper rash, tinea infections, etc., are various types of superficial fungal infections, subcutaneous fungal infection in tissues under the skin and lastly it causes systemic infection in deeper tissues. Most superficial and subcutaneous fungal infections are easily diagnosed and readily amenable to treatment. Opportunistic fungal infections are those that cause diseases exclusively in immunocompromised individuals, e.g., aspergillosis, zygomycosis, etc. Systemic infections can be life-threatening and are associated with high morbidity and mortality. Because diagnosis is difficult and the causative agent is often confirmed only at autopsy, the exact incidence of systemic infections is difficult to determine. The most frequently encountered pathogens are *Candida albicans* and *Aspergillus* spp. But other fungi such as non-albicans *Candida* spp. are increasingly important.

A majority of children, at one time or other suffers some or the other form of fungal infection. For instance, if a child develops a rash on the buttocks or white patches in the mouth, it is as a result of fungal or yeast infection.[1,2] Fungal infections which were quite rare at the beginning of this century are now increasingly growing at a rapid rate. This is probably due to the result of the increase in number of immunocompromised children.[3] Normally children have strong natural immunity to fungi. Only a couple of fungi out of thousands are pathogenic. Fungi are very good at taking advantage of some abnormality in the human host and, thus, virtually every fungal infection is opportunistic.[3–5]

Fungi are eukaryotic, nonmotile, and usually aerobic. They can exist as parasites or free living organisms and need organic sources of nourishment. They have a dense rigid cell wall made of glucan and chitin (found in crab shells). Their cell membrane contains sterols (ergosterol), making them similar enough to human cell membranes to have negative implications for the membrane destroying properties of antifungal drugs.[6,7]

Fungi come in many forms but only three are of our interest as they may cause disease in human being:

1. Yeasts – round/oval, unicellular, and reproduce via budding
2. Molds – long, floppy, fluffy colonies that microscopically can be seen as long tubular structures called hyphae and reproduce by forming spore-forming structures at the end of hyphae called conidia.
3. Dimorphs – most medically important, can change from yeast to mold and back, and grow in environment as molds and in humans as yeast. Fungi can produce toxins but this is not relevant to human infections. Fungi can produce human disease because of their sheer size (50–100 times larger than bacteria) and by eliciting an immune response as a result of themselves or their by-products.[8–9]

## Types of Fungal Infections

Fungal infections in children are broadly classified into three types:

- Superficial/cutaneous – present on skin, hair, nails
- Subcutaneous – infection in tissues under the skin
- Systemic – they are of two types: “true pathogens” (term is becoming obsolete) – which have the ability to cause disease in healthy hosts and opportunists – which cause disease exclusively in immunocompromised individuals.[10,11]

The above three infections are discussed in detail below.

### Superficial fungal infections

These infections are common all over the world (but specifically based on geographic distribution) and are caused by fungi called dermatophytes, which produce keratinase. This allows them to metabolize and live on human keratin, i.e., on skin, nails, and hair. They cause inflammation superficially but cannot invade deeper into the dermis. The common superficial fungal infections caused by *Tinea* worm are body ring worm by *Tinea corporis*, athlete’s foot by *Tinea pedis*, etc.[4,5,8,10] Such infections are red, itchy, and scaly. The diagnosis and confirmation should be based on clinical observations. The procedure which is followed is to scrape the lesion and observed under the microscope, and in case of Wood’s lamp (UV) colonies will fluoresce.

Another important superficial infection is cause by the yeast *Malassezia furfur*.[12] It does not even get into the keratin – more superficial than the dermatophytes. It digests the top layer of lipids and affects adolescents and adults to cause the superficial infection called *Tinea versicolor* – round, hypo-, and sometimes hyperpigmented patches. It never penetrates into the skin but can, especially nosocomially, infect the blood by contaminating lipid IV solutions.

Some of the types of superficial fungal infections that occur frequently in children are discussed below.

#### Oral thrush

It is an infection of the yeast fungus, *Candida albicans*, which occurs on the surface of the tongue and inside the mucus of the cheeks. It appears as white patches known as “plaques” which resemble milk curds.[13–15] One way to distinguish candida plaques from milk curds is that if the surface of the plaque is scraped away, a sore and reddened area will be seen underneath, which may sometimes bleed. It occurs most commonly in babies, particularly in the first few weeks of life.[16–18] Outbreaks of thrush in older children may also be the result of an increased use of antibiotics and steroids, which disturbs the balance of microbes in the mouth. This causes an overgrowth of *Candida*, which results in thrush.[19,20]

The stratified squamous epithelium of the oral mucosa forms a continuous surface that protects the underlying tissues and functions as an impervious, mechanical barrier. The protection so provided is dependent on the degree of keratinization and the continuous desquamation or shedding of epithelial cells. Indeed, the latter mechanism is considered to play a pivotal role in maintaining a healthy oral mucosa and in limiting Candidal colonization and infection. The interaction between *Candida* spp. and the commensal microbial flora is perhaps the next critical mechanism modulating oral Candidal colonization. The commensal flora regulates yeast numbers by inhibiting the adherence of yeasts to oral surfaces by competing for sites of adherence as well as for the available nutrients.

#### Candida diaper rash

It is sometimes called napkin dermatitis, a rash which occurs in the buttocks. Nappy rash will occur when the skin is sensitive and there is a presence of a trigger factor which includes prolonged exposure to urine and being unwell or thrush.[21–23] It tends to be in the deepest part of the creases in the groin and buttocks. The rash is usually red with a clearly defined border and consists of small red spots close to the large patches. In general, any diaper rash that lasts for 3 days or longer may be candidiasis.[24–26] A *Candida* skin infection can come from the upper gastrointestinal tract, the lower gastrointestinal tract, or exposure from a care provider. A *Candida* diaper rash can be accompanied by *Candida* infection of the mouth (thrush). A breastfeeding infant with a thrush infection may inadvertently infect the mother’s nipple/areola area. If such an infection is suspected, simple topical medications may be prescribed by her doctor.[27,28]

#### Tinea infection (ringworm)

It is a skin infection caused by fungi. It is called “ringworm” because the infection may produce ring-shaped patches on the skin that have red, wavy, worm-like borders. Some of the ways of catching Tinea is by direct skin-to-skin contact with an infected person, by sharing items with an infected person, or by touching a contaminated surface (such as floors in shower and locker rooms).[4]

Some of the more common types of Tinea infections that occur in children are discussed next. *Tinea capitis* results in a diffuse, itchy, scaling of the scalp that resembles dandruff. It can cause patches of hair loss on the scalp. It is especially common among children aged 3–9, particularly children who live in crowded conditions in urban areas. Scalp ringworm spreads via contaminated combs, brushes, hats, and pillows.[8,9] Tinea corporis means “ringworm of the body”; it involves the nonhairy skin of the face, trunk, arms, or legs. This would produce the classic ring-shaped patches with worm-like borders which may occur singly or in groups of threes and fours. It can occur in persons of all ages.[8]

*Tinea pedis* (athlete’s foot) produces area of redness, scaling, or cracked skin on the feet, especially between the toes. The affected skin may itch or burn, and the feet may have a strong odor. It is often acquired by walking barefoot on contaminated floors.[5]

*Tinea versicolor* or more commonly known as “white spots” is caused by a fungus known as *Malassezia furfur*. This fungus is present on the skin of most of the people but will only cause infection in some of them. This infection is common round the year in hot and humid climate. It occurs more often in older children and young adults.[10]

**Figure 1:**
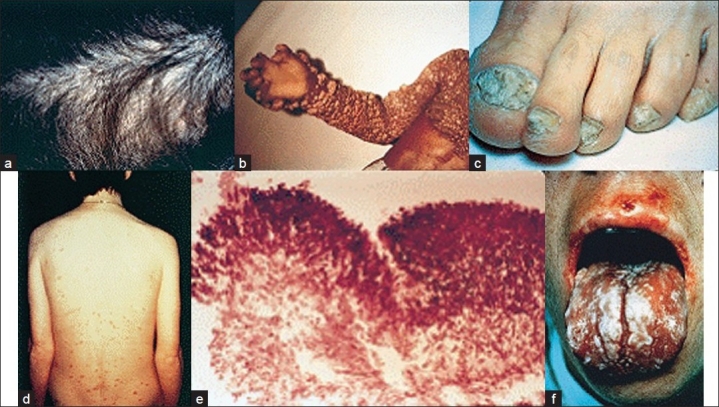
Clinical presentations of some frequently observed fungal infections: (a) *Tinea capitis* due to *Trichophyton tonsurans;* (b) onychomycosis due to Trichophyton rubrum; (c) chronic oral candidiasis; (d) chromoblastomycosis; (e) histopathological appearance of an aspergilloma. (f) Cutaneous lesions in a patient with disseminate candidiasis.[8] (Reproduced with permission from Richardson *et al*.)[29

The infection causes a rash which may appear on the back, neck, upper chest, shoulders, armpits, and upper arms. The skin rash consists of peeling, oval patches with sharply defined borders, and pimple-like bumps. The patches appear white or black on dark-skinned people and are usually pink or tan on the more fair-skinned. More often than not, it does not cause itching unless the person is hot or sweaty. The patches may be more prominent after the skin has been exposed to the sun, because the patches do not tan [Figure 1].[11]

### Subcutaneous fungal infections

If get a chance to introduce through the human skin, these fungi have the biological ability to grow in subcutaneous tissue and sometimes can cause significant human disease. They can grow up lymphatic and into bones or joints if in the way. These infections are far more common in the developed world. There are a variety of species that cause them: in India, Africa, and the Far East, generally speaking, they cause mycetoma (Madura foot). Individuals walking barefoot may get a splinter or injury to the foot and be inoculated, and slowly the bones of the foot and the ankle joint can be destroyed.[30]

Different types of subcutaneous fungal infection are discussed next.

#### Sporotrichosis

This infection is caused by the dimorphic fungus *Sporothriz schenkii*, which is characterized by the development on the skin, in subcutaneous tissue and in lymph nodes, of nodules which soften and break down to form indolent ulcers. The *fungus* is a saprophyte found widely on plants, thorns, and timber. Infection is acquired through thorn pricks or other minor injuries. Rare instances of transmission from patients *and infected* horses and rats have been recorded. It needs a splinter or a thorn to introduce it into human tissue – it can only live inside the body. It travels along lymphatics and causes a combination of pyogenic and granulomatous reaction. It manifests itself as a set of ulcerating nodules along a hard cord as it slowly grows up the lymphatics. It moves from distal to proximal and can lead to bone and joint destruction. It can become disseminated almost exclusively in immunocompromised individuals. The classic clinical presentation of this is with individuals with outdoor encounters: gardeners, golfers, hunters, etc. The culture mount of this fungus show fine branching hyphae and pear-shaped conidia borne in rosette like clusters at tips at lateral branches and singly along sides of hyphae.[31,32]

#### Chromoblastomycosis

This infection is caused by soil-inhabiting fungi of family Dematiaceae family. They enter the skin by traumatic implantation. The lesion develops slowly around the site of implantation. The most common fungi responsible are the *Fonsecaea* spp. – *F. pedrosoi*, F. *compactum*, *F. dermatitidis, Phialophora* spp – *P. verrucosa* and *Clostridium* spp. – *C. carrionii*. Infections caused by *F. pedrosoi* and *P. verrucosa* have been reported to disseminate to other areas, especially brain. Its clinical manifestations are raised and crusted lesions of the skin. Histological, the lesions show the presence of the fungus as round or irregular, dark brown, yeast-like bodies with septate called sclerotic cells, which can be diagnosed in KOH mounts or in sections and by culture on Sabouraud’s agar.[33]

#### Chronic mucocutaneous candidiasis

It is caused by *Candida* spp. (predominantly *C. albicans*). Various manifestations include white fissures, lesions, hyperkeratotic, granulomatous, and vegetative lesions, autosomal recessive trait associated with endocrine disorders, e.g., hypoparathyroidism.[34,35]

### Systemic fungal infections

After superficial and subcutaneous comes systemic fungal infection. These are less common but more serious. They can be divided broadly into two types namely (a) endemic infections and (b) opportunistic infection.

(a) *Endemic infections* infect all types of people, including those with a normal immune system. Although very much like TB, they cause disease only in specific circumstances. A huge number of individuals are infected, but only a few get diseased. As these infections have a number of properties in common, we should think of them as a group: histoplasmosis, coccioidomycosis, and blastmycosis.[36] Such infections are caused by dimorphs. They grow as molds in soil and reproduce there by sporulation. When they enter in a human, which happens exclusively via the respiratory route, they become yeasts. They also have a restricted geographic distribution and finally, and most importantly, they all cause disease with symptoms almost indistinguishable from TB.[37]

Various types of endemic fungal infections are elaborated next.

*Histoplasmosis*. It is an intracellular infection of the reticuloendothelial system caused by dimorphic fungus *Histoplasma capsulatum*. This fungus reacts like soils with high nitrogen content so it is found in the Ohio-Mississippi Valley, the Caribbean, and Central and South America. It is particularly endemic to chicken coups and caves due to the nitrogen in bird and bat droppings. It is caused by inhaling spores, which change into yeast in the lungs, phagocytosed by macrophages, and are disseminated hematogenously. After about 6 weeks, one can be tested for histoplasmin antigen derivative (just like TB), which is useful for diagnosing exposure but worthless for diagnosing disease. Clinically, histoplasmosis also mimics TB; almost everyone who is infected with this organism has latent disease. Very few people get sick, unless there is something else going on and can cause problems for those with an immature immune system or immunocompromised.[38] For people with abnormal lungs, histoplasmosis can cause pneumonia if inhaled into nonperfused areas. It can cause a chronic, cavitating nodular infection very similar to TB. Histoplasmosis can remain latent and reactivate many years later, for example, with AIDS. This can then disseminate and cause disease in virtually every organ of the body. To differentiate between TB and histoplasmosis, one can use sputum or blood smears, and purified protein derivative (PPD).[39]

*Coccioidomycosis*. This is an infection caused by fungus *Coccidiodes immitis*. This fungus is probably the most virulent and prefers to live in hot dry weather (Arizona, Cali, Mexico, Central, and South America). Infection is acquired by inhalation of dust containing arthrophores of the fungus. This fungus is dimorphic, occurring in the tissue as yeast and in culture as the mycelial form. The tissue form a spherule, 15–75 *μ*m in diameter, with a thick doubly refractile wall and filled with endospores. Unlike histoplasmosis, this dimorph turns into a spherule not yeast in the human lung. A spherule is a giant seedpod full of thousands of yeast particles called endosperms; thus, it can infect very efficiently (explains virulence). There is also a skin test for Cocci called coccidiodin. Like histoplasmosis, in this case spores are aerosolized and inhaled. When compared the sequence of events in TB and histoplasmosis, it was found that they are almost similar. However, unlike TB and histoplasmosis, the primary infection with Cocci can have symptoms because one is getting such a huge load of organisms, with the endospores disseminating in the lungs. One gets a self-limited, flu-like syndrome (sometimes called Bali fever). However, in some people it does disseminate (more commonly in pregnant women, immunocompromised, and darker skinned people) and causes skin, bone, and CNS disease.[40]

*Blastomycosis*. The fungus *Blastomyces dermatiditis* causes this disease. This fungus is a dimorph that lives in a soil as a mold and becomes yeast in the human being upon inhalation. It likes organic debris and humidity: woodlands, beaver dams, marshes, and peanut farms (for some unknown reason) and are usually found at mid-Atlantic, Carolinas, and Mississippi Valley. It is picked up from aerosolized spores. In its yeast form, blastomyces is much bigger than histoplasma and has broad-based buds and a small capsule. The pathogenesis is basically the same as the others. The one big difference is that no one has yet discovered a characteristic protein for a skin test. We usually do not have any idea how many people have been exposed and how many to develop disease with blastomyces. We do know that with a fraction of people (immuncompromised), the disease can go on to become disseminated or can stay in the lungs to cause a TB-like disease. It likes cool surfaces so it tends to cause a lot of skin disease (skin lesions that look and behave like skin cancer), bone disease, and urinary tract disease in men.[36,39]

(b) *Opportunistic systemic fungal infections* occur primarily when some aspect of the normal host defense is compromised. Such infections are life-threatening and are associated with high rates of death. Because of the growing population of immunocompromised individuals, the frequency of systemic fungal infections is increasing significantly.[40] Patients with hematological malignancies, such as myeloid and lymphocytic leukemias, are at particularly high risk of infectious complications. Infections can occur when the patient develops neutropenia of long duration caused by the hematological malignancy itself or the treatment administered. For example, patients receiving bone marrow transplants are given immunosuppressive drugs (e.g., corticosteroids or cyclosporine) to prevent or treat rejection. Intensive myelosuppressive chemotherapy also causes neutropenia in patients.

Approximately 20–50% of patients who die from hematological malignancies have evidence of invasive fungal infections at autopsy. The death rate is even higher in patients with mixed Aspergillus and Candida tissue infections. Solid organ transplant recipients who receive immunosuppressive medications to limit the risk of rejection also have an increased susceptibility to systemic fungal infections. Fungal infections occur in 5–45% of all solid organ transplant patients and are a primary cause of morbidity and mortality.[41,42] Burnt patients are another population at high risk; the wound site is susceptible to colonization by opportunistic fungi such as Candida, but nowadays this is generally well managed and Candidiasis in burnt patients may originate in the gastrointestinal tract or from intravenous catheters.[43] Changes in medical procedures have contributed to the increased incidence of systemic fungal infections. The skin and mucosal surfaces normally prevent micro-organisms entering the body, but, if these barriers are compromised, for example, during surgery or when in dwelling catheters are used, fungal cells are able to invade. Myelosuppressive chemotherapy may also cause mucosal lesions, allowing the invasion of fungi. Allogeneic bone marrow transplant recipients face the additional risk of developing graft-versus-host disease, which can damage the mucosal surfaces and normally requires an increase in immunosuppressive therapy.

In lung transplant recipients, the donor organ can also be a reservoir for pathogenic fungi, which may have been dormant or harmless in the donor but can be a source of post-transplantation infection in the immunosuppressed recipient. Previous fungal infection may predispose high-risk patients to subsequent systemic infections because fungi can remain dormant for some time and become reactivated when the patient is immunosuppressed.[44,45] High-risk patients with damaged mucosal surfaces caused by bacterial or viral infections may be at a higher risk of infection by *Candida* spp. A high level of colonization by Candida in the gastrointestinal tract and oral cavity may also increase the threat of systemic Candidal infection in patients at high risk. An additional contributory factor is the use of antibiotics, which disrupt the microbial flora, allowing colonization by fungi. Antibiotic therapy also leads to increased survival of patients who are predisposed to fungal infection. Immunocompromised patients readily acquire fungal infections from their environment if standard safety procedures are not followed. For example, *Aspergillus* spp. is airborne fungi and may be propagated through ventilation systems. The risk of infection can be greatly reduced by the use of high-efficiency particulate air (HEPA) filtration.[46] Transmission of Candida from the hands of medical professionals is also an important cause of nosocomial infections. Hand washing is therefore vital, but unfortunately the rigorous compliance necessary to reduce infection is not always present.[47] Other patients at risk include those with medical conditions that compromise the immune system, the most notable being advanced HIV immune dysfunction or AIDS. Because of the extreme degree of immunosuppression in the patient group, fungal infections can be unusually virulent and persistent. Between 60% and 90% of individuals with progressive HIV disease develop at least one fungal infection during the course of the disease.[48] Among the most frequently occurring fungal infections in this group are candidiasis, cryptococcosis, and less frequently aspergillosis,[49] although, in specific geographical regions, endemic mycoses are important (e.g., coccidioidomycosis, penicilliosis with *Penicillium marneffei*, and histoplasmosis).[50] Individuals with chronic granulomatous disease (CGD), an inherited abnormality of the neutrophils that serve as an important defense against fungi, are also predisposed to infection. Various types of opportunistic systemic fungal infections are described next.

*Invasive candidiasis/candidaemia*. It is caused by *C. albicans* and other nonalbicans *Candida* spp. Its clinical manifestations are prolonged antibiotic-resistant fever, often associated with weight loss, abdominal pain, and hepatic and/or spleen enlargement. CT scan may reveal small radiolucent lesions in liver or spleen in patients with chronic invasive candidiasis (hepatosplenic candidiasis).[55^56^]

*Invasive aspergillosis*. It is caused by *Aspergillus* spp. The commonest human disease caused by aspergilli is otomycosis. Its clinical manifestations are prolonged antibiotic-resistant fever, histopathological appearance of nonpigmented, septate hyphae with dichotomous branching. CT scan shows characteristic halo and/or air crescent signs. In this, the fungus actively invades the lung tissue. Disseminated aspergillosis involving the brain, kidney, and other organs in fatal complication sometimes seen in debilitated patients on prolonged treatment with antibiotic, steroids, and cytotoxic drugs.

Diagnosis may be made by microscopic examination and by culture. The fungus grows rapidly on culture media. Identification of *Aspergillus* is easy based on growth characteristics and morphology. *Aspergillus* has septate hyphae. Asexual conidia are arranged in chains, carried on elongated cells called “sterigmata,” borne on expanded ends (vesicles) of conidiophores.[51] Meningitis is the most common clinical manifestation. Hematogenous spread may also occur, giving rise to widespread cutaneous lesions.[52]

*Zygomycosis*. It is caused by *Rhizopus* spp., *Absidia* spp., and *Mucor* spp. It may manifest as rhinocerebral, pulmonary, gastrointestinal, or cutaneous mucormycosis. Disseminated mucormycosis can follow, spreading most frequently to the brain, with possible metastatic lesions in the spleen, heart, and other organs.

*Other invasive infections*. It is caused by *Malassezia* spp. Its manifestations are characterized by catheter-associated sepsis related to hyperalimentation with lipid emulsions and pneumonia. *Trichosporon* spp. can cause infrequent disseminated disease which occurs in granulocytopenic patients, with fatal outcome. Bloodstream infections manifest as skin and lung lesions and give false positives in cryptococcal antigen tests. Infections due to *Fusarium* spp. can cause mycetoma, endophthalmitis, facial granuloma, osteomyelitis, and brain abscess. Disseminated disease in neutropenic patients has positive blood cultures and skin lesions.[52]

### Diagnosis of systemic fungal infections

Diagnosis of systemic fungal infections is problematical and many infections are confirmed only at autopsy. The clinical symptoms are nonspecific and similar to those of bacterial and viral infections. In addition, the isolation of fungi from clinical samples is unreliable and may be complicated by the presence of a colonizing commensal organism, or ubiquitous fungi in the environment, causing false-positive results. Furthermore, the collection of clinical samples often requires an invasive procedure, which may not be advisable in critically ill patients. Direct microscopic examination of clinical samples may provide a tentative diagnosis, but this is often difficult to confirm by culture because of the presence of atypical fungal elements or sparse fungal populations. Serological tests that detect antibodies are low in sensitivity and specificity because many patients with systemic fungal infections are immunocompromised and, therefore, have an impaired antibody response. However, even in immunocompetent individuals, the delay between the onset of infection and the development of the antibody response reduces the practical value of these tests. Improvements in the diagnosis of invasive pulmonary aspergillosis include the greater use of routine high-resolution computed tomography (CT) scanning,[53] polymerase chain reaction (PCR) for the detection of RNA or DNA,[54] and enzyme-linked immunosorbent assay (ELISA) testing for circulating galactomannan, a component of the fungal cell wall in aspergillus.[55] None of these tests has been proven to determine definitive invasive disease but positive results are generally highly predictive of invasive disease. Sequential positive results from laboratory diagnostic tests may be the best means of predicting systemic fungal infection; however, because of the life-threatening nature of the condition, there is usually not sufficient time to obtain a definitive diagnosis of the causative agent before treatment is required.

Another complicating factor when considering appropriate antifungal therapy is that some patients are infected by more than one pathogen simultaneously.[55]

### Prevention Tips

The chances of children getting fungal infections can be minimized by making sure that they practice good hygiene. If possible, children are not allowed to share personal items such as brushes and bath towels. It should be ensured that they do not go bare-footed at public places, such as shower rooms and swimming pools. And finally, after bathing, children should be towel-dried to prevent any tinea infections.

### Conclusion

The incidence of local and systemic fungal infections is increasing at an alarming rate. This is primarily due to advances in medical practice, which have resulted in the proliferation of severely ill, immunocompromised, hospitalized patients. The HIV epidemic and other diseases of the immune system have added to this growing at-risk population. Superficial fungal infections are mild but may spread to other areas of the body or, occasionally, to other individuals. More seriously, but less frequently, they may develop into invasive forms. Although not life-threatening, conditions such as onychomycosis can have a severe impact on a patient’s quality of life and self-image. It is therefore desirable to treat local fungal infections to prevent the risk of spread. Systemic fungal infections in immunocompromised patients, such as bone marrow and solid organ transplant recipients, are associated with high rates of mortality. *C. albicans* and *Aspergillus* spp. are the most prevalent pathogens in systemic infections, but a recent shift in the burden of disease has seen the emergence of a number of important non-albicans *Candida* spp. in addition to rare infectious agents such as *M. furfur*. Although the incidence of fungal infections can be reduced by minimizing risk factors in hospitals, such as poor hygiene practice, our inability to reliably prevent such infections highlights the need for effective treatments.
